# Preventing food contamination: preliminary results of a cross-sectional study among food handlers

**DOI:** 10.1093/eurpub/ckac130.103

**Published:** 2022-10-25

**Authors:** EA Errico, F Licata, R Maruca, N Costantino, G Di Giuseppe, F Napolitano, CP Pelullo, G Della Polla, S Angelillo, A Bianco

**Affiliations:** 1 Department of Health Sciences, University of Catanzaro ‘‘Magna Græcia, Catanzaro, Italy; 2 Department of Experimental Medicine, University of Campania “Luigi Vanvitelli”, Naples, Italy; 3 Department of Movement Sciences and Wellbeing, University of Naples, Naples, Italy; 4 Health Direction, Teaching Hospital, University of Campania “Luigi Vanvitelli”, Naples, Italy

## Abstract

**Background:**

More than 600 million people around the world get sick every year due to eating contaminated food, which is impressive considering that all foodborne diseases (FDs) are preventable. Contamination during food preparation by food handlers (FHs) is one of the main causes of FDs. The aim of this study is to assess the knowledge and behaviors of FHs related to FDs.

**Methods:**

The cross-sectional study was conducted in two regions (i.e. Calabria and Sicily) of Southern Italy. Data was collected through an anonymous self-administered questionnaire designed to retrieve sociodemographic information, knowledge about food safety, and food-handling behaviors among a randomly selected sample of FHs ≥ 18 years of age.

**Results:**

Findings refer to a sample of 171 respondents with a mean age of 40 years (SD ± 12.7). A vast majority (63.7%) of FHs did not know the correct procedure for hand washing according to Food & Drug Administration (FDA) guidelines and 28.4% of subjects did not wash their hands after touching raw food, which constitutes a major risk of food cross-contamination. One-third of FHs were unknowledgeable that cross-contamination (e.g. using the same utensils for cooked and raw foods) could lead to FDs. Multiple logistic regression analysis showed a positive correlation (p < 0.001) among good knowledge and proper food-handling practices, such as using separate kitchen utensils to prepare cooked and raw foods and storing them in separate areas or fridges. Less than half of the sample (42.1%) reported the need for more information about FDs.

**Conclusions:**

Preliminary results highlight a lack of knowledge about simple rules to avoid food cross-contamination that could negatively impact on food safety and food-handling behaviors. This study add evidence about areas where intervention are needed to reduce the occurrence of FDs.

**Key messages:**

- Incorporating knowledge and behavioral-based food safety training programs could be effective to control the risk of FDs.
- Further research is needed on strategies to improve behavior change.

